# THE PERICAPSULAR NERVE GROUP (PENG) BLOCK IN PHYSICAL AND REHABILITATION MEDICINE: AN OPINION PAPER

**DOI:** 10.2340/jrm.v58.46559

**Published:** 2026-09-22

**Authors:** Simon BUTET, François GENET, Charles GUIGNANS, Antoine GEFFRIER, Isabelle BONAN

**Affiliations:** 1 Physical Medicine and Rehabilitation Department, CHU Rennes, Rennes; 2 UPOH (Unité Péri Opératoire du Handicap, Perioperative Disability Unit), Department of PMR “Suivi au long cours”, Raymond-Poincaré Hospital, GHU Paris-Saclay, Assistance Publique – Hôpitaux de Paris (AP-HP), Garches; 3 Versailles Saint-Quentin-en-Yvelines University (UVSQ – Paris-SaclayParis-Saclay University), UR 20262 Handistart, UFR Simone Veil Santé, Montigny-le-Bretonneux; 4 Orthopedic Surgery Department, CHU Rennes, Rennes; 5 University Rennes, INRIA, CNRS, INSERM, IRISA, EMPENN ERL U1228, Rennes, France

First described in 2018, the pericapsular nerve group block (PENG block) has emerged as a significant advancement in the management of hip pain within perioperative anaesthetic practice (1). The technique rapidly gained adoption due to its simplicity and, under ultrasound guidance, its ability to selectively reach the articular sensory branches of the femoral and accessory obturator nerves, which together supply the majority of sensory innervation to the anterior hip capsule (2). In anaesthesiology, a growing number of randomized controlled trials consistently demonstrate that the PENG block provides effective perioperative analgesia (3). These studies highlight the block’s ability to reduce opioid consumption and preserve motor function. The characteristics of the PENG block, with a selective sensory and motor-sparing profile (no or low impact on quadriceps strength), make it particularly relevant for functional contexts in which pain directly limits mobility and recovery. This perspective is especially pertinent in Physical and Rehabilitation Medicine (PRM), where pain management is inseparable from functional goals. PENG block can be regarded not only as an analgesic technique but also as a functional tool enabling rehabilitation.

Since our first report on the effectiveness of the PENG block (4), we have performed over 50 procedures in our department and believe it would be useful to share our experience in PRM.

## WHY DOES THE PENG BLOCK MATTER FOR PRM?

In neurological or musculoskeletal rehabilitation settings, chronic hip pain, most often associated with degenerative conditions, is a frequent reason for PRM consultation, and its prevalence continues to rise with population ageing (5, 6). Moreover, increased life expectancy of persons with disabilities, combined with the preservation of walking ability despite persistent lower-limb malalignment, has resulted in a growing number of people developing both primary and secondary degenerative hip disease.

Hip pain assessment in PRM could be challenging, for example in patients presenting with overlapping spine or referred knee pain. The diagnostic ambiguity is exacerbated in people with neurological disorders by spasticity, cognitive impairment, and different pain mechanisms involving nociceptive, neuropathic, and nociplastic components. The PENG block provides a minimally invasive diagnostic tool for clarifying the origin of hip-related pain.

Moreover, in such patients, PENG is a highly desirable therapeutic tool, as it relieves pain by temporarily reducing nociceptive input while preserving motor function, thereby supporting mobility restoration and improving participation, especially when incorporated into a rehabilitation programme. Although total hip arthroplasty is now technically feasible in most patients with neurological disabilities (7), its indication is often delayed by the decline in general condition of these patients and the availability of specialized centres with expertise in implant selection, surgical management, and postoperative rehabilitation. In this context, the PENG block could serve as a bridge to surgery or as a palliative alternative when surgery is not an option. For all these reasons, the technique warrants broader awareness and dissemination within the PMR community.

## HOW CAN PRM PHYSICIANS PERFORM A PENG BLOCK?

### Anatomical and ultrasound considerations

Sensory innervation of the hip capsule primarily comes from the femoral, obturator, and accessory obturator nerves. Additional branches originate from the nerve to the quadratus femoris, the superior gluteal nerve, and the sciatic nerve, especially for the posterior capsule (8). The anterior capsule receives the densest sensory innervation, particularly in its anterior and superolateral regions, which contain the highest concentration of mechanoreceptors and sensory fibres (9, 10). The hip joint also receives vegetative innervation mainly from the peri-arterial (adventitial) nerve plexus (which does not pass through the PENG injection plane) and is involved in the trophic regulation of capsuloligamentous tissues as well as in synovial secretory activity and bone remodelling.

Ultrasound visualization of the key landmarks for accessing the sensory branches of the anterior hip (femoral and accessory obturator) is generally easy: the antero-inferior iliac spine, the iliopubic eminence, and the iliopsoas tendon. Identification of the obturator branch, either via a subpectineal approach (11) or targeting the inferomedial acetabular region at the radiographic “teardrop” (12), may be more challenging, as approaches targeting the posterior capsule (13) are not necessarily essential in our experience.

### Choosing the anaesthetic agent

Most publications on the PENG block originate from the field of regional anaesthesia, relying predominantly on local anaesthetics such as lidocaine or ropivacaine in perioperative contexts (3). From a rehabilitation perspective, the analgesic benefit of local anaesthetics may persist well beyond their pharmacological duration (a few hours), probably through interruption of nociceptive input, reduction of central sensitization (14), and restoration of movement through rehabilitation. Within PRM, lidocaine-based PENG blocks can serve as a valuable diagnostic test to predict the potential benefit of therapeutic interventions. Ropivacaine, which has a longer-lasting effect, should be preferred when PENG is used to facilitate mobilization and help patients “break through a plateau” during a rehabilitation programme. Corticosteroid adjuvants could be useful, and some teams combine ropivacaine with dexamethasone for extended analgesic effect with encouraging clinical outcomes (15).

### Injection volume considerations (local anaesthetic PENG block)

Volumes reported in the literature vary widely (approximately from 5 to 20 mL), with no consensus regarding the optimal volume. Diffusivity of the injected solution and ultrasound accuracy must be considered. Excessive diffusion may affect the motor branch of the femoral nerve (quadriceps) or the anterior branch of the obturator nerve (mainly adductor longus), posing a risk of transient weakness. Cadaveric studies suggest that 10 mL seems to be sufficient to obtain a “true” pericapsular block, limiting unnecessary diffusion (16).

### Hip deformity considerations

Performing a PENG block may be challenging in patients with advanced hip deformities such as fixed flexion or adduction contractures, obesity, previous hip surgeries leading to altered local anatomy, or neurogenic heterotopic ossifications. These situations, often seen among neuro-orthopaedic patients, can compromise ultrasound visualization and needle access. In our opinion, recent hip radiography should therefore be obtained before performing a PENG block. Probe adjustments, optimized positioning, and low-frequency transducers may help. When ultrasound-guided localization remains difficult, fluoroscopic guidance in the operating room, potentially under general anaesthesia, should be considered, relying on the established anatomical landmarks commonly used for radiofrequency neuromodulation procedures (12).

### Potential functional risks considerations

PENG block may occasionally induce transient motor weakness due to diffusion (17), potentially affecting knee stability and increasing fall risk during early mobilization. In terms of sensitivity, analgesia may transiently mask protective pain signals and disturb the patient’s sensations. Neuropathic complications after PENG block appear to be rare in the current literature, although transient nerve irritation remains a theoretical risk common to regional anaesthesia techniques. Consequently, post-procedure nursing monitoring is required and should include assessment of quadriceps strength, vigilance during transfers, and ambulation until motor function is fully confirmed.

## BEYOND LOCAL ANAESTHETICS: CHEMICAL HIP DENERVATION THROUGH THE PENG APPROACH

Although the PENG block was initially developed as a regional anaesthetic technique, the same ultrasound-guided anatomical approach can also be used to perform selective chemical denervation of the hip by targeting the articular sensory branches with neurolytic agents. This emerging application opens new therapeutic perspectives for prolonged pain relief in degenerative hip disorders such as osteoarthritis or avascular necrosis (18, 19).

Neurolytic agents include absolute ethanol and glycerol–phenol solutions. Both induce selective neurolysis through protein denaturation, leading primarily to demyelination and Wallerian degeneration of large myelinated fibres, as demonstrated histologically and electrophysiologically (20). Their predictable, concentration-dependent neurolytic effect has been used for decades in PRM, mainly for motor nerve neurolysis in the management of spasticity (21).

More recently, the PENG approach has extended the use of these agents to the sensory articular branches of the hip. Since 2018, several case reports have described ultrasound-guided alcohol neurolysis for inoperable hip fractures (22) and metastatic hip pain (23), while more recent studies have explored this technique for degenerative hip disorders (18, 19). In 2025, our group reported the first PRM case of ultrasound-guided phenol hip denervation in a young woman with neurological disability, resulting in sustained pain relief and gait improvement (4).

Contrary to motor neurolysis, chemical denervation of the sensory innervation of the hip raises specific procedural considerations. At the PENG injection site, neurolytic agents target the terminal articular branches of the femoral and accessory obturator nerves, which do not provide cutaneous innervation. Consequently, the theoretical risk of deafferentation pain or cutaneous sensory loss is expected to be low. To date, we have not observed neuropathic pain following this procedure in our clinical experience. Absolute ethanol typically induces a transient burning pain during injection, whereas phenol exerts an immediate local anaesthetic effect before producing concentration-dependent neurolysis (21), better tolerated in terms of pain. Furthermore, glycerol–phenol formulations are more viscous and may limit unwanted spread. Based on our preliminary experience, we currently favour ultrasound-guided injection of a sterile 7% (weight/volume) phenol in glycerol solution (10 mL) for hip denervation through the PENG approach.

However, intentional sensory denervation also raises important questions in rehabilitation medicine. While prolonged analgesia may facilitate function and participation, reducing protective nociceptive feedback could theoretically increase the risk of joint overuse and neurogenic arthropathy. Further studies are therefore needed to better define the long-term safety of chemical hip denervation.

## CHEMICAL DENERVATION OR RADIOFREQUENCY ABLATION?

An alternative to chemical neurolysis is radiofrequency ablation (RFA). Its mechanism involves thermal lesioning (continuous or cooled RF) or neuromodulation (pulsed RF) of the sensory branches of the obturator and femoral nerves (± accessory obturator) near the anteromedial hip capsule. This technique has mainly been studied in non-neurological participants with osteoarthritis (24), ineligible for arthroplasty or with persistent post-arthroplasty pain. RFA requires costly equipment, fluoroscopic expertise, and often per-procedural sedation. Anatomical variability of articular branches may complicate precise targeting. Conversely, the PENG approach benefits from the natural diffusion of injection, which helps compensate for micro-variations in nerve trajectory and achieves wide denervation of the anterior capsule (16). Neurolytic agents are more accessible and are already part of routine practice in many PRM centres with a modest learning curve for physiatrists accustomed to ultrasound guidance. Duration of effect comparison remains difficult to determine due to variable concentrations and volumes used in existing studies. No randomized controlled trial has yet been published comparing chemical denervation with radiofrequency ablation. An ongoing randomized trial is currently addressing this question for the hip (ClinicalTrials.gov – NCT06570746), while the RADIOPHENOL trial (25) has recently adopted a similar comparative approach for genicular nerve denervation, reflecting the growing interest in these new therapeutic approaches.

## INDICATIONS AND POSITIONING WITHIN THE PRM THERAPEUTIC ARSENAL

In Physical and Rehabilitation Medicine, the PENG block is particularly useful in hip degenerative conditions for managing hip pain while preserving function. It serves as a diagnostic aid, a facilitator of rehabilitation, a temporizing option before surgery, or as treatment for patients who are non-operative and at risk of functional decline.

### Focus on neuro-orthopaedic considerations

The PENG block appears particularly relevant in the field of orthopaedic surgery for people with nervous system lesions, which encompasses patients with central or peripheral neurological disorders, including congenital, genetic, and traumatic conditions, leading to complex neuro-orthopaedic joint deformations (26). Chronic hip pain is common in this population and can result in significant loss of autonomy. Its pathophysiology is often multifactorial, involving hip dysplasia, altered biomechanics of the hip, referred pain, and, in many cases, spastic paresis with imbalance between paretic and hypertonic muscles, which exacerbate pain and contribute to a vicious circle of functional decline. In these complex neuro-orthopaedic situations, therapeutic decision-making remains challenging due to a delicate risk–benefit balance. Hip pain management should be tailored to the patient’s functional status and surgical eligibility. Treatment should prioritize conservative and targeted approaches, including physiotherapy, analgesics, injections, spasticity management, and, when appropriate, total hip arthroplasty performed by specialized multidisciplinary teams with expertise in complex neuromuscular hip disorders (27). When arthroplasty is contraindicated, PENG block may represent a valuable therapeutic alternative, potentially avoiding more invasive procedures, such as femoral head–neck resection, associated with significant morbidity.

Neuro-orthopaedic reasoning relies on a multimodal approach combining clinical examination, functional assessment, and targeted interventional procedures to better characterize pain generators and guide treatment strategies. Within this decision-making framework, diagnostic nerve blocks already play a central role by helping to differentiate the respective contributions of spasticity and soft tissue contractures. In this regard, the PENG block integrates naturally as a complementary diagnostic tool, specifically targeting the sensory innervation of the anterior hip capsule, and thereby refining the identification of articular nociceptive sources. Beyond its diagnostic contribution, the PENG block also fits coherently within the therapeutic tools of neuro-orthopaedics. It complements other PRM interventions, including botulinum toxin injections, chemical neurolysis, or cryoneurolysis (28), to reduce pain and achieve functional goals.

## FUTURE DIRECTIONS

Further evaluation of PENG block techniques is essential to define their precise role within structured, PRM-specific decision-making algorithms for chronic hip pain. A comparable interventional approach may also be considered at the knee. Genicular nerve denervation is emerging as a promising option for chronic knee pain management (29). Prospective comparative studies should extend beyond analgesic outcomes to include functional goals such as walking, transfers, participation in therapy, nursing care, and caregiver burden, reflecting the core priorities of rehabilitation practice. Importantly, the PENG block aligns with the functional logic of PRM, offering pain relief and the possibility of active rehabilitation. Its adoption within PRM requires dedicated training for physicians, supporting both technical proficiency and appropriate patient selection. Its integration has the potential to enhance the procedural toolkit of rehabilitation specialists.
